# The effects of synthetic orally administrated insulin nanoparticles in comparison to injectable insulin on the renal function markers of type 1- diabetic rats

**DOI:** 10.22038/ijbms.2020.42292.9985

**Published:** 2020-06

**Authors:** Nejat Kheiripour, Behnam Alipoor, Akram Ranjbar, Yasin Pourfarjam, Farzaneh Kazemi Najafabadi, Narges Dehkhodaei, Masoumeh Farhadiannezhad, Hassan Ghasemi

**Affiliations:** 1 Research Center for Biochemistry and Nutrition in Metabolic Diseases, Kashan University of Medical Sciences, Kashan, Iran; 2 Department of Laboratory Sciences, Faculty of Paramedicine, Yasuj University of Medical Sciences, Yasuj, Iran; 3 Toxicology and Pharmacology Department, School of Pharmacy, Hamadan University of Medical Sciences, Hamadan, Iran; 4 Department of Chemistry, University of Cincinnati, Cincinnati, OH, United States of America; 5 Student Research Committee, Abadan School of Medical Sciences, Abadan, Iran; 6 Department of Clinical Biochemistry, Abadan Faculty of Medical Sciences, Abadan, Iran

**Keywords:** Chitosan, Diabetes mellitus, Insulin nanoparticle, Kidney injury molecule 1, Neutrophil gelatinase-associated lipocalin protein

## Abstract

**Objective(s):**

Injectable insulin is the most widely used therapy in patients with type 1 diabetes which has several disadvantages. The present study was aimed to evaluate the efficacy of injectable insulin on diabetes mellitus-related complications in comparison to orally encapsulated insulin nanoparticles.

**Materials and Methods:**

This study involved 42 Wistar rats separated into 5 groups, including control (C), diabetic control (D), diabetic receiving regular insulin (INS), diabetic receiving encapsulated insulin nanoparticle (INP), and diabetic receiving chitosan for two months. Biochemical parameters in serum and urine were measured using spectrophotometric or ELISA methods. mRNA levels of kidney injury molecule 1 (KIM-1) and neutrophil gelatinase-associated lipocalin (NGAL) were evaluated using quantitative PCR.

**Results:**

There were no significant differences between the two forms of insulin in controlling the glycemic condition (*P*-value>0.05), but oral INP was more effective in correcting diabetic dyslipidemia in comparison to injectable insulin (*P*-value<0.05). Urine volume and creatinine excretion were significantly modulated by insulin and oral INP in diabetic groups (*P*-value<0.05), although the effects of INP on the modulation of execration of urea, acid uric, and albumin was more dramatic. Oral INP caused a significant decrease in urine concentration of KIM-1 and NGAL as well as expression of KIM-1 in renal tissue (*P*-value<0.05).

**Conclusion:**

Our results suggested that oral INP is more effective than injectable insulin in modulation of urine and serum diabetic-related parameters.

## Introduction

Diabetes mellitus (DM) is a metabolic disorder defined by hyperglycemia and impaired metabolism of macromolecules (1). According to a report by the international diabetes federation (IDF), more than 400 million people (20–79 years old) suffered from DM in 2017 and this number is expected to go beyond 600 million by 2045 (2). Ethologically, DM is categorized as type 1 diabetes (T1D), largely resulting from autoimmune destruction of insulin-producing β-cells and defect in insulin production, and type 2 Diabetes (T2D) which may be due to insulin resistance in peripheral tissues such as muscle and adipose. Generally, chronic clinical complications of DM are divided into macrovascular complications such as cardiovascular disease (CVD), and microvascular complications

including nephropathy, retinopathy, and neuropathy (3). There are several mechanisms involved in hyperglycemia-induced DM complications which include induction of polyol pathway, diacylglycerol (DAG)/protein kinase C (PKC) signaling pathway, free radicals production, stimulation of advanced glycation end products (AGE) formation, and activation of hexosamine pathway (3). Previous results showed that hyperglycemia disrupts vascular homeostasis mainly through inhibitory effects on several factors including vascular endothelial growth factor (VEGF), platelet-derived growth factor (PDGF), activated protein C (APC), and insulin (4).

One of the major complications of DM that may lead to end stage renal disease (ESRD) is diabetic kidney disease (DKD) (5, 6). Previous evidence revealed that approximately 30% of patients with T1D and 40% of patients with T2D suffered from this complication (7). In general, hyperglycemia induces renal damage by different mechanisms such as change in glomerulus hemodynamic, defect in endothelial cell function, changes in the basal membrane structure, and podocyte injury (8). There are several markers for evaluation of DKD; Kidney injury molecule 1 (KIM-1) and Neutrophil gelatinase-associated lipocalin (NGAL) are among the new markers, which based on the Food and Drug Administration (FDA) reports might be appropriate for kidney injury evaluation (9, 10). 

Insulin therapy is the most common method for glycemic control in T1D patients and some of the T2D patients (11). Subcutaneous injection is one of the routine methods for insulin delivery. However, this method has some disadvantages such as pain at the injection site, lipoatrophy, trauma, skin hypertrophy, increased risk of infection, as well as aversion to injection in public places (12, 13). It is thought that oral administration of insulin not only overcomes these limitations, but also it may mimic the physiological conditions in a better way in comparison to subcutaneous injection (14). This strategy, nevertheless, has two challenging issues: first, destructive effects of gastrointestinal tract on the orally administrated insulin, and second, the low permeability of insulin across the cell membrane due to its size and its hydrophobicity properties (15).

One of the promising strategies in this context is to encapsulate insulin into carriers in order to improve its delivery efficacy and its bioavailability (16-18). Nanoparticles are appropriate candidates among carriers that are applied by several pharmaceutical companies. Chitosan is a linear cationic heteropolysaccharide composed of D-glucosamine and N-acetyl-D-glucosamine derived from deacetylation of chitin (19). Chitosan is among the most widely used carriers in drug delivery due to its interesting properties such as high biocompatibility, high biodegradability, non-toxicity effects as well as high stability (20, 21). Furthermore, cationic chitosan is capable of interacting with anionic glycoproteins on intestinal epithelial cells membrane, thereby passing more easily through cell tight junctions (22).

Given the importance of DKD evaluation as one of the main complication of DM, and improving the efficiency of insulin delivery, we studied the effects of orally administrated encapsulated insulin nanoparticles on different parameters in serum and urine of T1D rats in comparison to injectable insulin.

## Materials and Methods

***Insulin nanoparticle synthesis***

*Synthesis of quaternized N-aryl derivatives of chitosan*

Briefly, 3 g of chitosan powder (Primex) was added to 300 ml of acetic acid 1% and mixed properly to make a jelly solution. After adjustment of pH to 5.5 by NaOH 1N, 2.5 g of 4-N,N-dimethyl Benz aldehyde (Merck) was added to the solution and then mixed to produce a phosphorus green suspension. The procedure continued by adding NaOH to the suspension, separation of sediment and then washing by methanol. Afterward, 2 g of dried sediment was mixed with 70 ml of N-methyl pyrrolidone (Merck) and heated at 100 ^°^C for 48 hr. After this step, 1 gr potassium iodide was added and then heated at 50 ^°^C following by adding 2 ml of NaOH 1N and 12 ml of methyl Iodide. Finally, after heating the mixture for 24 hr at 50 ^°^C, 2 liters of acetone were added, the sediment then was separated using a Buchner funnel and dried. Furthermore, characterization of quaternized derivatives was evaluated by H-NMR. 

*Loading insulin into nanoparticles*

Insulin was loaded into nanoparticles by modified polyelectrolyte complexation (PEC) method (23). In this method, 0.03 g of quaternized and aromatized chitosan polymer produced in the previous step was added to 30 ml of acetic acid 1% to produce a chitosan polymer solution. Insulin with negative charge was generated by mixing 0.03 g of lyophilized insulin (Exir Pharmaceutical, Iran) in 30 ml acetic acid followed by adjustment of pH to 8.2. The insulin solution was added (flow of 1 ml/min) to chitosan solution, mixed and then centrifuged at 17000 rpm for 20 min to produce insulin nanoparticles (INPs) in the sediment phase. The supernatant phase was used for evaluation of loading and entrapment efficiency. 

*Evaluation of physical properties of INPs*

Different properties of INPs including size, polydispersity, and zeta potential were evaluated by Zetasizer ZEN3600 (Malvern instrument, UK) at 25 ^°^C. Morphology of INPs was assessed using Transmission Electron Microscope (TEM). 

*Measurement of loading and entrapment efficiency*

The loading efficiency (LE) and entrapment efficiency (EE) were studied by high performance liquid chromatography (HPLC) (LC 20AD XR, Shimadzu) equipped with a vacuum degasser, C18 (250 mm×4.6 mm, 5 μm) column, and UV-VIS photodiode array detector. Twenty-five microliters of the samples was injected to HPLC and the mixture of acetonitrile and sodium sulfate (1.5:1 v/v respectively) was used as mobile phase at 1 ml/min flow rate for 5 min at 25 ^°^C. Loading efficiency and entrapment efficiency were measured based on the following equation (23): 

LE%=Total amount of insulin-Free amount of insulin Weight of nanoparticles×100

EE%=Total concentraion of insulin-Free concentration of insulin Total concentraion of insulin×100

*Lyophilization and encapsulation of INPs*

INPs were dissolved in mannitol 5% and then placed in a freeze dryer Lyotrap Plus (LTE Scientific Ltd, Oldham, UK) at -46 ^°^C and 0.07 mbar pressure for 48 hr. Finally, lyophilized INPs were encapsulated by preclinical capsules (Capsugel, Belgium).

*Coating of INPs*

Prepared INPs were coated by Eudragit L100 (Merck, Germany). In this method, 20 g of Eudragit L100 powder was added to NaOH 1N and stirred for 5 days to produce a jelly solution. Afterward, preclinical capsules filled by lyophilized INPs were added to this solution and then dried. Coating of the encapsulated insulin nanoparticle with Eudragit L100 led to release of INPs only in the jejunum and protected it against the destructive effects of the stomach. 

***Evaluation of insulin release***

Simulated gastric fluid (SGF) and simulated intestinal fluid (SIF) were used for *in vitro* release assessment of nanoparticles in the stomach and intestine, respectively. SGF was prepared by adding hydrochloric acid solution (0.2 N, 39 ml) to sodium chloride solution (0.2 N, 250 ml), and the volume was then adjusted to 1000 ml with deionized water and pH was maintained at 2.2. SIF was provided by dissolving KH_2_PO_4_ (6.8 g) in 250 ml of deionized water. The solution was mixed with NaOH (0.2 N, 77 ml) and then adjusted to 1000 ml using deionized water, while the pH was maintained at 6.8. Encapsulated INPs coated by Eudragit L100 were added into SGF medium and after 15, 30, 45, 60, 75, 90, and 120 min, 1 ml solution was sampled from SGF and analyzed by HPLC. Finally, encapsulated INPs were transferred from SGF to SIF and sampling was repeated and insulin release was measured by HPLC. 

***Animals and study design***

Forty-two adult male Wistar rats (214–244 g) were involved in this study. They were kept under standard conditions (12-hr dark/light cycle at 22±2 ^°^C). The animals were randomly divided into 5 groups: Group C: normal control treated with normal saline (n=8), group D: diabetic control (n=10), D+INS: diabetic group treated with regular insulin (5 u/kg daily, subcutaneous injection) (Exir Pharmaceutical Company, Iran) (n=8) (24), D+INP: diabetic group treated with capsulated INPs (30 u/kg daily, orally) (n=8), and D+ Chitosan: diabetic group treated with chitosan (15 mg/kg, by gavage) (n=8). The treatment course was 60 days. The induction of T1D was done by streptozotocin (STZ, Sigma) (60 mg/kg; subcutaneous injection) dissolved in citrate buffer (0.1 M, pH: 4.5). Induction of T1D was confirmed after 72 hr by measurement of FBS using a glucometer (Accuchek; Roche, Germany). Rats whose FBS levels were beyond 250 mg/dl were regarded as diabetic. At the end of treatment, the animals were transferred to metabolic cages to collect 24-hr urine then anesthetized by ketamine (50 mg/kg). After collecting blood samples (from vena cava) rats then were sacrificed. Kidney tissues were separated, and liquid nitrogen was used to freeze and save them at -70 °C. The Medical Ethics Committee of Abadan Faculty of Medical Sciences approved all procedures of the current study (IR. ABADANUMS.REC. 1395.84)

***Lipid profile***

Measurement of the rats’ lipid profile including triglycerides (TG), total cholesterol TC, and high density lipoprotein cholesterol (HDL-C) was done by a kit (Pars Azmun, Iran) while low density lipoprotein cholesterol (LDL-C) was calculated using the Friedewald formula.

***Measurement of biochemical parameters***

The concentrations of urea, uric acid, creatinine, and albumin (Alb) were assayed using a kit (Pars Azmun, Iran).

***Measurement of NGAL and KIM-1 in urine and plasma***

The NGAL and KIM-1 concentrations in plasma and urine were assayed using an ELISA kit (Cusabio Biotech, Wuhan, China), according to the manufacturer’s instructions.

***Measurement of KIM-1 and NGAL expression***

Total RNA extraction of renal tissues was done by RNX- Plus reagent (Cinnagen, Tehran, Iran). Afterward, the Prime Script RT reagent kit (TaKaRa Biotechnology, Japan) was used to synthesize complementary DNA (cDNA) according to the manufacturer’s instructions. In order to perform quantitative Real-Time PCR reaction, SYBR premix Ex TaqTM II (TaKaRa Biotechnology, Japan) was employed using a Roche Light Cycler 96 System (Roche Life Science Deutschland GmbH, Sandhofer, Germany). Amplification conditions included initial denaturation in 95 ^°^С for 30 sec followed by 30 cycles (95 ^°^С for 20 sec, 58 ^°^С for 30 sec, and 72 ^°^С for 30 sec). β-actin gene was used to normalize relative gene expression. The forward and reverse primers included: NGAL, forward: 5’-GATGAACTGAAGGAGCGATTC-3’, reverse: 5’-TCGGTGG GAACAGAGAAAAC-3’, KIM-1, forward: 5’-ACTCCTGCAGACTGGAATGG-3’, reverse: 5’-ACTCCTGCAGACTGGAATGG-3’, β-actin, forward: 5’-TCA TTG ACC TCA ACT ACA-3’, and reverse: 5’- CAAAGTTGTCATGGA TGACC- 3’. The relative gene expression was calculated by the 2^-ΔΔCt^ method.

***Statistical analysis***

Data were analyzed using SPSS software version 16.0 (SPSS Inc., Chicago, USA) and the graph was prepared using Graph Pad Prism version 6.0 (Graph Pad Software, San Diego, USA). All data were shown as mean±SD. To compare the mean of variables, one-way analysis of variance (ANOVA) was used followed by Tukey’s test. Significance level was set at *P*<0.05.

## Results

***H-NMR spectrum of quaternized aromatized chitosan***

**Figure1:**
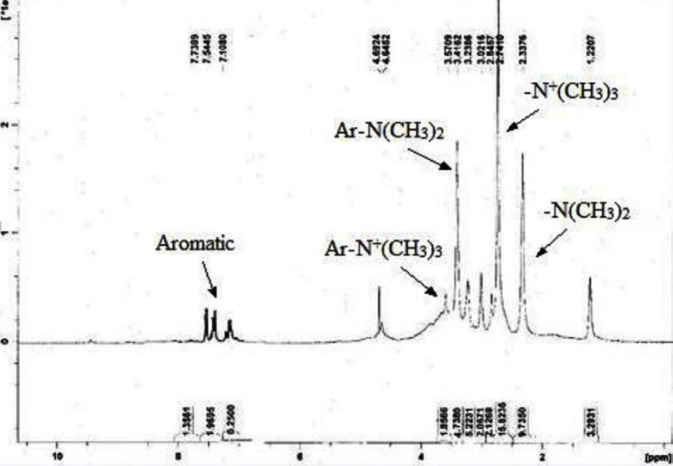
H-NMR spectra of quaternized aromatized chitosan

The H-NMR spectrum of quaternized aromatized chitosan is shown in Figure 1. Peaks at 2.3 and 2.7 ppm are related to the protons of the methyl groups of [-N(CH_3_)_2_] and [-N^+^(CH_3_)_3_] in methylated aliphatic amine. Furthermore, peaks at 3.4 and 3.6 ppm represent the protons of the methyl groups of [-N(CH_3_)_2_] and [-N^+^(CH_3_)_3_] in aromatic amine attached to benzyl ring. 

***Characteristics of INPs***

The size, zeta potential, and polydispersity of INPs before and after lyophilization are presented in Table 1. Results showed that after lyophilization, these parameters increased in comparison to before the lyophilization.

**Figure2:**
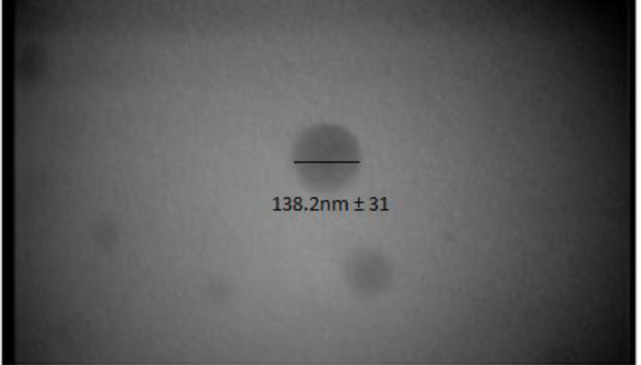
Evaluation of encapsulated insulin nanoparticles by transmission electron microscope (TEM)

The LE and EE of INPs were 17.96%±0.45 and 62.03±0.22, respectively. Evaluation of shape by TEM in different magnifications confirmed the spherical shape of INPs (Figure 2). 

***Release of insulin in SGF and SIF***

**Figure 3:**
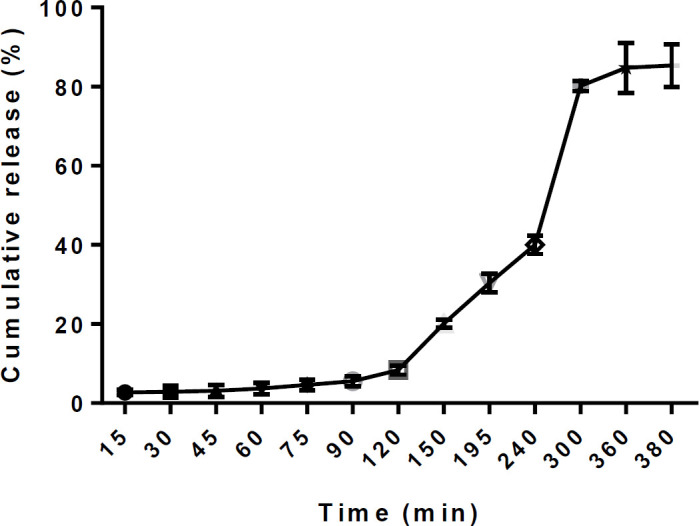
Release of insulin from encapsulated insulin nanoparticles coated by Eudragit L100. Release of insulin in SGF (0-120 min) was 8.35% and this amount was 84.81% in SIF (120-360 min)

Figure 3 shows the results of *in vitro* release assessment. The amount of insulin released during 120 min in SGF was 8.3%. However, this amount was 84.81% during 120–360 min in the SIF medium. Therefore it can be concluded that coating with Eudragit L100 has been effective in releasing insulin into the jejunum. 

***General and biochemical parameters***

The results of general and biochemical measurements are presented in Table 2. Compared with the control group, the final body weight significantly decreased in the diabetic group (*P*-value<0.05). Treatment with insulin and encapsulated INPs significantly improved final body weight (*P*-value<0.05). Induction of diabetes by STZ caused significant increase in FBS (*P*-value<0.05), and insulin treatment in the diabetic group as well as encapsulated INPs significantly ameliorated the blood glucose (*P*-value<0.05). However, FBS in diabetic groups that received insulin and encapsulated INPs was still significantly higher than that for the control group (*P*-value<0.05).

Furthermore, STZ-induced T1D significantly increased TG and LDL-C concentrations compared with the control group (*P*-value<0.05), but insulin treatment and encapsulated INP remarkably modulated the levels of LDL-C (*P*-value<0.05). STZ-induced diabetes significantly increased TG levels compared with the controls (*P*-value<0.05), and treatment with insulin and encapsulated INPs did not have a significant effect on this level (*P*-value>0.05). Compared with the control group, the concentration of HDL-C in diabetic group and diabetic groups that received insulin was significantly lower (*P*-value<0.05), but no significant difference was observed between diabetic groups that were treated with encapsulated INP and the control group (*P*-value>0.05). 

Compared with the control group, the concentrations of urea and creatinine in the diabetic group and diabetic that received insulin group experienced a significant increase (*P*-value<0.05). However, encapsulated INPs significantly improved urea concentration (*P*-value<0.05). T1D caused a significant increase in uric acid (*P*-value<0.05), and treatment by insulin and encapsulated INPs modulated it significantly (*P*-value<0.05). Finally, chitosan was found to have no significant effects on body weight and biochemical parameters (*P*-value>0.05).

***Measurement of urine parameters***

Table 3 indicates the results of urine parameters assay. Compared with the control group, urine volume in treated and untreated diabetic groups had a significant increase (*P*-value<0.05). However, treatment of diabetic groups with insulin and encapsulated INPs significantly decreased urine volume (*P*-value<0.05). T1D significantly increased concentration of urine urea, uric acid, and creatinine (*P*-value<0.05), and treatment by insulin and encapsulated INPs significantly decreased these parameters (*P*-value<0.05). The effect of encapsulated INPs on the reduction of uric acid levels was significant in comparison to insulin (*P*-value<0.05). Although T1D significantly increased excretion of albumin (*P*-value<0.05), treatment with encapsulated INPs improved it (*P*-value<0.05). There were no significant differences between diabetic groups and diabetic groups treated with chitosan (*P*-value>0.05). Treatment with chitosan has no significant effects on the urine parameters (*P*-value>0.05).

***Gene expression of KIM-1 and NGAL***

**Figure 4:**
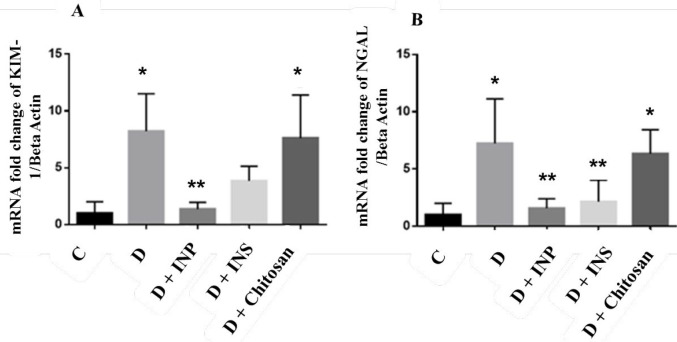
The effect of injectable insulin and encapsulated insulin nanoparticles on the gene expression of kidney injury molecule 1 (KIM-1) (a) and neutrophil gelatinase-associated lipocalin (NGAL) (b) in studied groups. The data are presented as mean±SD. C, healthy control; D, diabetic control; INP, encapsulated insulin nanoparticles (30 U/kg); INS, insulin (5 U/kg daily); chitosan (15 U/kg). * Significantly different compared with C groups. ** Significantly different compared with D groups, *(P*-value<0.05)

Figure 4 presents the results of gene expression of KIM-1 and NGAL in renal tissue (A and B, respectively). There was a significant increase in the expression of KIM-1 in the diabetic group and diabetic group that received chitosan compared with the controls (*P*-value<0.05). Encapsulated INPs in diabetic groups significantly decreased KIM-1 expression (*P*-value<0.05). The NGAL gene expression also was significantly higher in diabetic groups and treated groups with chitosan compared with controls, however, treatment with encapsulated INPs and insulin in diabetic groups significantly decreased it (*P*-value<0.05).

***Concentration of KIM-1 and NGAL in urine and plasma***

**Figure 5:**
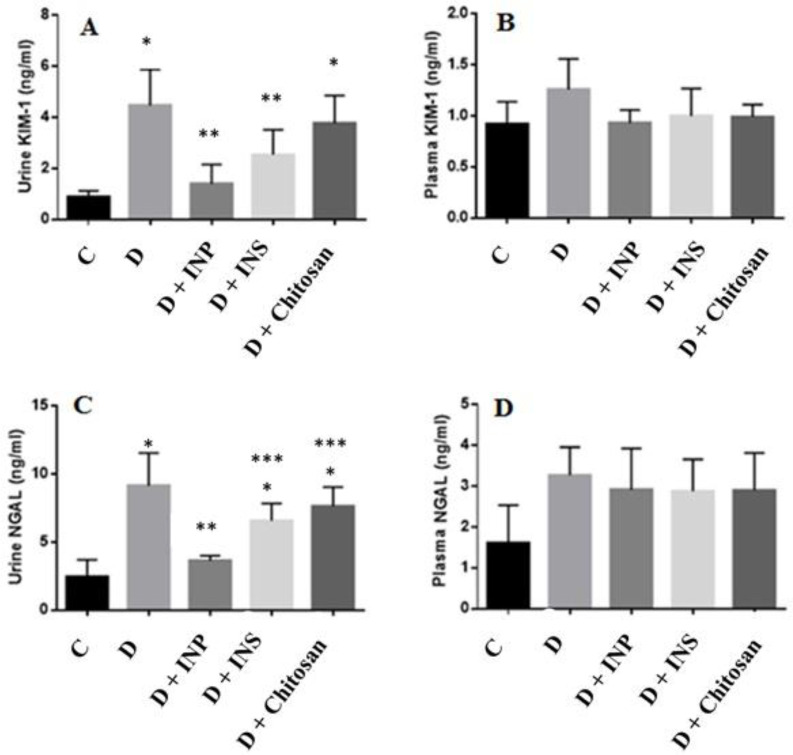
The effect of injectable insulin and encapsulated insulin nanoparticles on the (a) urine kidney injury molecule 1 (KIM-1), (b) plasma KIM-1, (c) urine neutrophil gelatinase-associated lipocalin (NGAL) and (d) plasma NGAL. Data are presented as mean±SD. C, healthy control; D, diabetic control; INP, encapsulated insulin nanoparticles (30 U/kg); INS, insulin (5 U/kg daily); chitosan (15 U/kg). * Significantly different compared with C groups. ** Significantly different compared with D groups. *** Significantly different compared with D+INP groups, (*P*-value<0.05)

 The results of KIM-1 and NGAL measurement in urine and plasma are presented in Figure 5. STZ-induced T1D caused significant increase in urinary KIM-1 concentration (*P*-value<0.05), treatment with encapsulated INPs as well as insulin, however, remarkably decreased its level (*P*-value<0.05) (Figure 5A). There was no significant difference between groups for plasma concentration of KIM-1(*P*-value>0.05) (Figure 5B). Compared with the controls, the urine levels of NGAL in diabetic groups and diabetic groups that received chitosan and insulin were significantly higher (*P*-value<0.05). However, encapsulated INPs significantly decreased it (*P*-value<0.05) (Figure 5C). No significant difference was found across the studied groups in terms of plasma concentration of NGAL (*P*-value>0.05) (Figure 5D). 

## Discussion

DM is one of the main challenges in the current century and a costly burden on society. Injectable insulin is the best choice for treatment of T1D patients and some T2D patients. However, in addition to several side effects, this method of therapy has many psychological consequences for DM patients. Nowadays, nanotechnology plays an important role in the development of pharmaceutical strategies. The current study showed that encapsulated INPs, as well as insulin, had considerable effects on the maintenance of the body weight and glucose homeostasis. However, INPs were more effective in the correction of T1D-induced dyslipidemia and renal function. Furthermore, INPs had a considerable effect on the modulation of KIM-1, NGAL, and renal damage biomarkers, in comparison to injectable insulin.

According to the results of our study, STZ-induced diabetes decreased body weight. However, insulin and INPs counteract with this effect. The loss of weight under diabetic conditions has been revealed by previous studies (25, 26). Indeed, inability of cells to metabolize carbohydrates as a source of energy can induce lipolysis and proteolysis, which subsequently lead to weight loss in DM conditions (27). As shown in previous studies that are in agreement with our results, INPs, as well as insulin, could improve body weight in T1D rats (28, 29). Insulin promotes glucose uptake, synthesis of glycogen and proteins, and generally induces the anabolism pathways, therefore, the maintenance of body weight in diabetic rats that were treated by injectable insulin is not an unexpected issue. However, considerable effects of oral INPs in balancing body weight is a point worth mentioning. The physiochemical barriers of gastrointestinal tracts such as environmental pH, hydrolytic enzymes, and cell to cell junctions are the main factors that counteract with the passage of orally administrated insulin (24). However, in the current study, improvement of body weight of T1D rats that were treated with orally administrated INP highlights the success of our strategies for delivery of insulin. 

STZ caused a considerable increase in FBS, and treatment with insulin and INPs significantly decreased this level. STZ enters the pancreatic β-cells by glucose transporter 29 (GLuT29) and induces DNA alkylation that is followed by necrosis and degradation of β-cells. Our results showed that encapsulated INPs, as well as insulin, could decrease FBS in T1D rats. In agreement with our study, Heidarisasan *et al.* showed that oral INPs had considerable effects in reduction of FBS in T1D rats model (28). Jamshidi *et al.* also revealed that oral INPs can ameliorate T1D-induced hyperglycemia (29). The potential of oral INPs to decrease FBS in T1D rats confirms again the efficacy of chitosan nanoparticles in delivery of insulin. Chitosan is a polymer that enables forming an ionic interaction with the negatively charged biomolecules at the epithelial cells of the intestine (30). In addition, we used quaternary ammonium in the structure of chitosan nanoparticles, positively charged nanoparticles with high solubility in a wide range of pH therapy were formed (31), resulting in delivery of insulin with high efficacy. 

Our results revealed that STZ-induced T1D increased the concentrations of TC, TG, and LDL but decreased HDL-C concentrations. In other words, orally encapsulated INP was more effective in correcting dyslipidemia in comparison to injectable insulin. The association between T1D induced by STZ and lipid profile has been documented by previous studies. A study showed that T1D significantly increased TC and TG levels. The proposed mechanism is based on two points: first, the concentrations of chylomicron and LDL-C are increased due to insulin deficiency in T1D, and second, decreasing the activity of lipoprotein lipase in T1D condition which eventually leads to hypertriglyceridemia (32, 33). Therefore, treatment by exogenous insulin could correct lipid profiles in T1D patients. The metabolism of orally encapsulated INPs is different from injectable insulin in that it is transferred directly from the intestine to the liver, and based on the central role of the liver in metabolism of macromolecules, it is expected that insulin loaded into nanoparticles is more effective than injectable insulin (34). 

One of the important points about oral administration of insulin is the use of a formulation that prevents the release of insulin in the stomach. In this study, encapsulated INPs were coated by eudragit l100 polymer which dissolved in the jejunum (pH: 6-7) and thereby release of insulin in the small intestine was guaranteed. Evaluation of insulin release in the current study also confirmed this issue and showed that insulin is released considerably in SIF. Another important issue regarding the application of nanoparticles in drug delivery is the stability of nanoparticles. In this study, we used the lyophilization process to improve the stability of nanoparticles. On the other hand, the use of mannitol during the lyophilization process inhibits the accumulation of nanoparticles and degradation of insulin.

Furthermore, our findings showed that T1D caused increase in urea, uric acid, and creatinine, whereas treatment by insulin and INPs significantly decreased these parameters. In accordance with our results, results by other researchers revealed that treatment by encapsulated form of insulin as well as insulin decreased the serum concentration of urea (35). Increasing the levels of creatinine and urea in DM is related to inability of cells to use glucose in DM that is compensated by catabolism of proteins, amino acids, and phosphocreatine which results in production of high levels of urea and creatinine (36, 37). Insulin and INP decreased the urea and creatinine concentrations by inducing the uptake and metabolism of glucose and also decrease in the breakdown of proteins. Uric acid is the final product of purine nucleotide metabolism which acts as an antioxidant. High levels of uric acid in DM might result from elevated production or from excretion inability (38). Imbalance between production and degradation of free radicals which results in oxidative stress is one of the main factors that is involved in DM pathogenesis (39). Accordingly, one of the mechanisms that improved DM-induced oxidative damage is elevation of antioxidant levels. Taken together, it can be concluded that high levels of uric acid in T1D rats is a defense mechanism to overcome oxidative stress (40). Diabetic hyperglycemia disturbs the nicotinamide adenine dinucleotide NADH/NAD ratio and is one of the main factors involved in oxidative stress (41). Accordingly, administration of insulin might ameliorate the DM hyperglycemia and improve oxidative stress.

The urinary levels of creatinine, urea, uric acid, and Alb were completely increased in T1D rats and significantly decreased by insulin and INP administration. The effect of orally encapsulated INPs on the improvement of albuminuria was considerable. The high levels of urea, uric acid, and creatinine in the urine of T1D rats are due to increased production of these parameters. Diabetic hyperglycemia directly contributed to hypertension, change in hemodynamic, and change in base membrane structure of glomerular capillary, and thereby plays an important role in albuminuria (42). Therefore, insulin and INP correct albuminuria through improvement of glucose homeostasis and correction of hemodynamic parameters. 

In the current study, T1D increased the gene expression and concentrations of KIM-1 and NGAL proteins in urine. However, insulin and INP caused a significant decrease in KIM-1 and NGAL gene expression and the level of these proteins in urine. KIM-1, an extracellular protein, is overexpressed in T1D and is one of the biomarkers for evaluation of kidney damage (43). NGAL is also a ubiquitous lipocalin iron-carrying protein that is attached to gelatinase in specific granules of neutrophil and acts as a biomarker in ischemic renal injury and repair processes (44). In agreement with our results, it has been documented that kidney damage in DM increased the renal expression of KIM-1 and NGAL (45). We previously showed that gene expression and urinary levels of KIM-1 and NGAL significantly increase in T1D rats (46). As one important limitation in our study, histology of kidneys was not evaluated. However, another study revealed that hyperglycemia-induced by diabetes results in damage to the extracellular matrix that is followed by increased vascular permeability, impaired blood flow, ischemia and hypoxia, and subsequently diabetic nephropathy (47). It seems that T1D- induced hyperglycemia is the main cause of NGAL and KIM-1 up-regulation. Results of other researchers confirm this hypothesis. They showed that hyperglycemia by T1D is the main factor involved in NGAL overexpression (48). Not surprisingly, therefore, expression of NGAL and KIM-1 increase in T1D rats. However, the considerable effects of orally encapsulated INPs on expression of NGAL and KIM-1 genes compared with insulin is an important point that indicates the importance of nanoparticles in drug delivery in our study. Overexpression of NGAL and KIM-1 in urine, but not in plasma, highlights the potential of these proteins as the markers of T1D-induced renal damage. 

**Table 1:** Physical characteristics of insulin nanoparticle (INPs) before and after lyophilization

Parameters	Before lyophilization	After lyophilization
Size (nm)	138.2 ± 31	324.33 ± 22.94
Zeta potential	+9.31 ± 1.18	+13.01 ± 1.1
Polydispersity	0.243±0.07	0.345±0.05

**Table 2:** The effect of insulin and INP on the body weight and biochemical parameters in the serum of the studied groups

Variable/group	C	D	D+INP	D+INS	D+ Chitosan
Initial body weight (gr)	228.04±9.3	230.3±10.4	227.2±9.2	224.2±3.2	226.6±4.9
Final body weight (gr)	294.8±13.9	194.46.9 ^a^	232.3±6.7 ^a, b, c^	220.1±8.3 ^a, b, c^	199.02±7.1^a^
Pre-diabetic FBS (mg/dl)	87.1±7.9	85.3±8.3	85.9±8.1	81.3±9.4	77.5±3.3
Post-diabetic FBS (mg/dl)	84.5±5.3	321.04±21.2^a^	338.5±10.1^a^	328.04±18.7^a^	322.3±17.4^a^
Pre-treatment FBS (mg/dl)	82.06±8.4	407.5±14.6^ a^	395.8±31.9^ a^	387.1±19.2^ a^	397.5±17.1^ a^
Final FBS (mg/dl)	81.4±4.1	429.5±15.6^ a^	221.2±15.5^ a, b, c^	244±12.6^ a, b, c^	410.1±16.8^ a^
Total cholesterol (mg/dl)	94.5±4.4	150.4±10.6^ a^	129.2±11.2^ a, b^	135.8±12.1^ a^	145.1±11.6^ a^
TG (mg/dl)	80.6±9.1	152.1±11.9^ a^	132.9±15.04^ a^	140.9±9.7^ a^	147.06±7.6^ a^
LDL-C (mg/dl)	38.3±5.8	110.7±9.5^ a^	56.1±10.2^ b, d^	78.3±13.01^ a, b^	106.2±13.09^ a^
HDL-C (mg/dl)	51.4±9.06	31.06±8.06^ a^	39.6±6.01	34.72±7.1^a^	30.3±3.7^ a^
Creatinine (mg/dl)	0.39±0.16	2.1±0.74 ^a^	1.2±0.24	1.59±0.52^ a^	2.13±0.73^ a^
Urea (mg/dl)	21.1±5.4	48.6±7.8 ^a^	30.9±3.8^ b^^, c^	37.04±7.8 ^a^	47.6±6.2^ a^
Uric acid (mg/dl)	2.92±0.88	6.7±1.07^ a^	3.75±0.79^ b, c^	4.37±0.98^ b^	6.01±1.36^ a^

**Table 3:** The effect of insulin and INP on the urinary parameters of the studied groups

Variable/group	C	D	D+INP	D+INS	D+ Chitosan
Urine volume (ml/day)	12.8±3.9	52.8±6.3 ^a^	33.6±7.02 ^a, b, c^	37.2±6.1^a, b, c^	49.6±7.5^ a^
Creatinine (mg/day)	8.3±2.71	24.5±5.87^a^	13.08±1.79^ b^	15.6±3.36^ b^	20.52±5.08^ a^
Urea (g/day)	0.19±0.04	1.85±0.41^a^	0.51±0.07^ b, c^	0.87±0.1^a, b, c^	1.71±0.19^ a^
Uric acid (mg/day)	0.36±0.07	4.1±0.65^ a^	1.8±0.49^ a, b, c, d^	2.87±0.25^ a, b^	3.5±0.46^ a^
Albumin (mg/day)	3.9±1.27	13.5±3.4^ a^	7.5±3.4^ b^	9.5±2.2^a^	10.6±3.2^a^

Data are presented as mean±SD. C, healthy control; D, diabetic control; INP, encapsulated insulin nanoparticles (30 U/kg); chitosan (15 U/kg); INS, insulin (5 U/kg daily). a Significantly different compared with control groups. b Significantly different compared with diabetic control groups.c Significantly different compared with D+Chitosan groups. d Significantly different compared with D+INS. *P*-value<0.05

## Conclusion

To date, therapy by injectable insulin has been the best choice to overcome T1D complications. The important message of the current study is that orally administrated INPs have remarkable effects on improvement of body weight, glucose homeostasis, lipid profile, and other serum and renal markers in T1D rats in comparison to injectable insulin. However, administration of oral INPs for DM patients requires further investigation.
